# A preliminary study on the potential of Nanopore MinION and Illumina MiSeq 16S rRNA gene sequencing to characterize building-dust microbiomes

**DOI:** 10.1038/s41598-020-59771-0

**Published:** 2020-02-21

**Authors:** Anders B. Nygaard, Hege S. Tunsjø, Roger Meisal, Colin Charnock

**Affiliations:** 1 Faculty of Technology, Art and Design, Department of Civil Engineering and Energy Technology, Oslo Metropolitan University (OsloMet), Oslo, Norway; 2 Faculty of Health Sciences, Department of Life Sciences and Health, OsloMet, Oslo, Norway; 3 Møreforsking Ålesund AS, Ålesund, Norway

**Keywords:** Data processing, Environmental microbiology, Bacterial genes

## Abstract

There is a growing awareness of the importance of indoor microbiomes for human health. Given their complexity, these microbiomes can only be adequately surveyed using high throughput sequencing techniques. Oxford Nanopore’s MinION is the newest third generation sequencing technology on the market. With its many advantages such as portability, user friendliness, simplicity, speed of sequencing and long read length, the technology is now an actual contender to established sequencing platforms. MinION’s main disadvantage is a relatively low read accuracy compared to several other platforms, although this is constantly improving. The present study, which appears to be the first of its kind, provides the results of a preliminary analysis of the microbial communities in indoor environments based on 16S rRNA gene amplicon sequencing, using both the Oxford Nanopore Technologies (ONT) MinIOn and the Illumina MiSeq DNA sequencers. At the level of family and above, there was no significant difference between the microbial compositions as revealed by the two platforms. However, at the genus, and particularly at the species level, the ONT MinION reported greater taxonomic resolution than Illumina MiSeq.

## Introduction

Built environments impact human health and disease, especially in countries where people spend a major part of the day indoors^1^. The indoor microbiome originates from many different sources, such as the communities of microbes that reside in/on the human body, from building components such as plumbing and ventilation, as well as from outdoor environmental sources that are brought inside^2^. Studying the indoor microbiome may help us understand how the indoor environment affects human health^3–5^.

Several studies have investigated the taxonomic diversity of bacterial communities in dust samples from buildings^6–10^. Amplification of the 16S rRNA gene coupled with high-throughput sequencing (HTS), allows for deep investigations into microbial communities. Technological advances continue to drive down costs, making HTS affordable and available for use in a wide range of novel research areas. Although several sequencing platforms and standardized protocols are available for HTS analysis^11^, there are differences between them and results may, therefore, diverge. Illumina sequencing platforms producing very high quality, but short (~ 300 bp) reads have been widely employed in the field of 16S rRNA amplicon sequencing^11^. This approach only permits analysis of a sub region of the 16S rRNA gene and taxonomic assignment of reads at the species level may be elusive.

In 2015, Oxford Nanopore Technologies (ONT) made the ultraportable mobile phone-sized MinION platform based on the ONT single molecule sequencing technology commercially available. The nearly unrestricted read length possible with the MinION sequencer allows for sequencing of full-length 16S rRNA gene amplicons, albeit with a slightly lower per read accuracy than many other HTS platforms. Despite the higher error rate, the increased sequence length provided by MinION might make possible the identification of bacterial taxa to the species level^12^.

Although the potential of using the MinION platform to analyze the bacterial composition at the species level is promising, this has not been comprehensively explored. The major aim of the present study, although restricted to a relatively small number of samples, was to investigate if the ONT MinION sequencing platform might offer promise for investigating the structure of the microbiota in dust collected from kindergartens and nursing homes. We consider how long-read sequences (ca. 1400 bp) obtained from the MinIon sequencer compare to short-read sequences (ca. 300 bp) obtained from Illumina MiSeq for classification of bacteria present in the indoor environment.

## Results

### Generation of 16S rRNA gene amplicon sequences

Illumina 300-bp paired-end sequencing generated a total of 2203794 sequence reads, with on average 183650 sequence reads per dust sample. After quality filtering a total of 582032 sequence reads, with on average 48503 amplicon sequence variants (ASVs) per dust sample were kept for analysis (Table 1).

**Table 1:** Sequence reads generated per sample for both short-read and long-read amplicons.

Dust sample	Long-read (Nanopore MinION) sequences		Short-read (Illumina MiSeq) sequences	
	Basecalled reads	Quality filtered reads	Raw reads	Quality filtered ASVs
BC01	110876	54844	226871	65755
BC02	291500	139155	203113	53082
BC03	200113	98096	174566	41615
BC04	160369	75926	242435	83706
BC05	123811	60284	100713	9853
BC06	224674	106228	159852	43962
BC07	140339	69134	146955	37023
BC08	132054	66637	146309	36999
BC09	328163	159733	226871	55803
BC10	306439	141752	184965	50335
BC11	272927	126516	198860	56749
BC12	116811	58502	192284	47150
Average per sample	200673	96401	183650	48503
Total	2408076	1156807	2203794	582032

Sequencing of long-read 16S rRNA amplicons on Nanopore MinION generated a total of 2408076 sequence reads after basecalling, with on average 200673 sequence reads per sample. After quality filtering of the basecalled sequences, 1156807 sequence reads were retained with an average of 96401 sequence reads per sample (Table 1).

### Taxonomic assignment of 16S rRNA gene amplicon sequences

For the short-read sequences, 582032 ASVs were taxonomically assigned using vsearch against Greengenes (GG) and SILVA. The DADA2 pipeline uses an ASV approach where the sequences themselves function as the unique identifier for taxons, rather than grouping reads into operational taxonomic units (OTU). 1156751 long-read sequence reads were passed from quality control to taxonomic assignment and aligned using LAST against GG and SILVA. The full SILVA and Greengenes databases contain approximately 190 000 and 99 000 sequences, respectively^9^.

The degree of assignment of long and short read sequences at different taxonomic levels, obtained when using GG and SILVA reference databases, is shown in Table 2. With respect to short read sequences, SILVA achieved a higher degree of identification at all taxonomic levels. However, for long read amplicons there was more variation in the performance of the databases. SILVA performed better at the species level and GG was able to assign more taxa at the higher levels, particularly at the order level (Table 2).

**Table 2:** Taxonomic assignment of short-read (Illumina Miseq) and long-read (Nanopore MinION) amplicons against the Greengenes (GG) and SILVA 16S rRNA gene reference databases.

Amplicon libraries	Sequence reads	Reads assigned to taxa (%) using GG				Reads assigned to taxa (%) using SILVA			
		Order	Family	Genus	Species	Order	Family	Genus	Species
Short-read amplicons	582032	577498 (99.2%)	344192 (59.1%)	212154 (36.5%)	25160 (4.3%)	580750 (99.8%)	458093 (78.7%)	296079 (50.9%)	132237 (22.1%)
Long-read amplicons	1156751	1152320 (99.6%)	580097 (50.1%)	424382 (36.7%)	145786 (12.6%)	570007 (49.3%)	568994 (49.2%)	283501 (24.5%)	227096 (19.6%)

### Efficiency of taxonomic assignments based on long- and short-reads

When using GG, in total 732 taxa were identified at the species level based on long- and short-reads. Of these, 91.7% could only be assigned based on long-reads generated by the MinION platform (Table 3). When using SILVA, 10475 bacterial species were identified. Of these 99.5% were only found by analysis of long-read sequences.

**Table 3:** Number of taxa identified at the different taxonomic levels using GG and Silva.

Database	Greengenes 97%				SILVA 99%			
Level	Total	Shared	Nanopore only	Illumina only	Total	Shared	Nanopore only	Illumina only
Order	225	114 (50.7%)	93 (41.3%)	18 (8.0%)	260	127 (48.8%)	108 (41.5%)	25 (9.6%)
Family	303	192 (63.4%)	98 (32.3%)	13 (4.3%)	918	233 (25.4%)	652 (71.0%)	33 (3.6%)
Genus	930	257 (27.6%)	657 (70.6%)	16 (1.7%)	2122	530 (25.0%)	1499 (70.6%)	93 (4.4%)
Species	732	58 (7.9%)	671 (91.7%)	3 (0.4%)	10745	37 (0.3%)	10693 (99.5%)	15 (0.1%)

### Bacterial taxa in dust samples revealed by short and long-read 16S rRNA gene sequencing

**Figure 1:**
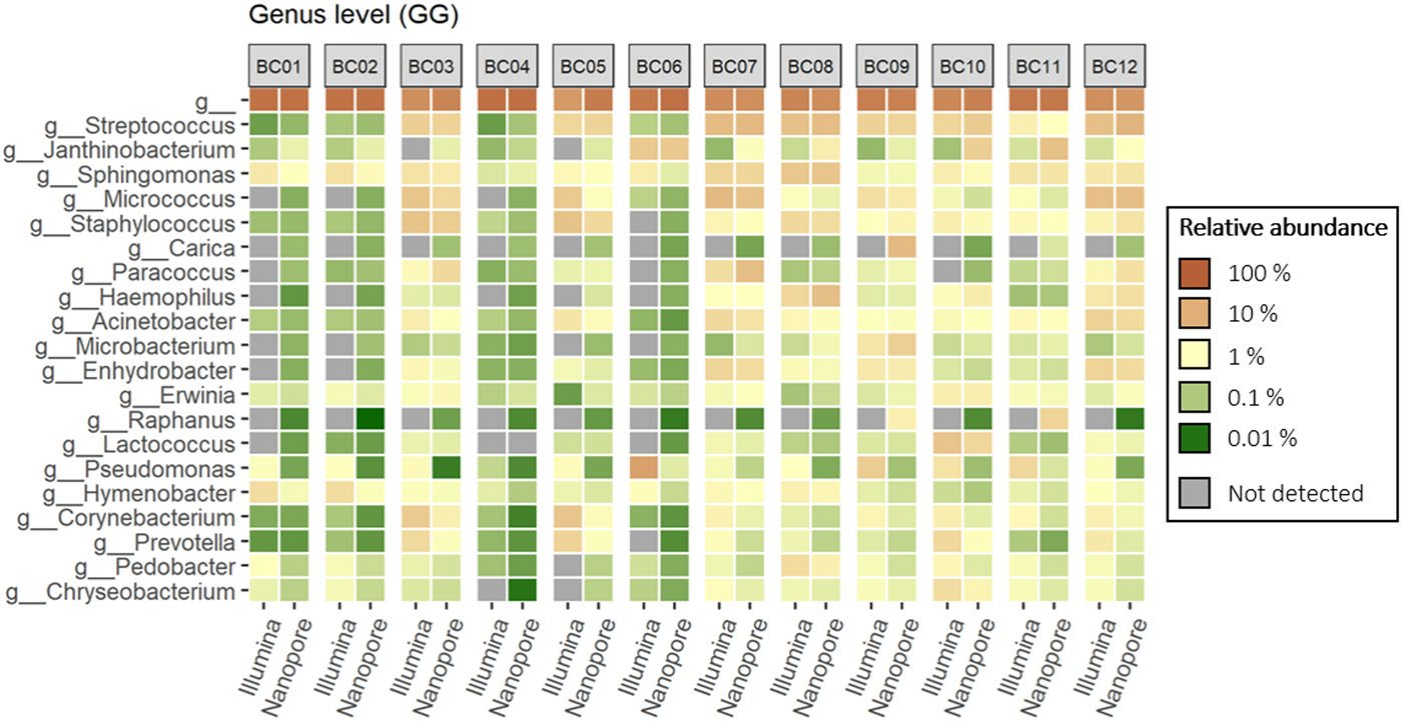
Heatmap of the 15 most abundant genera identified by mapping 16S rRNA gene amplicons sequenced on Illumina MiSeq and Nanopore MinION against the Greengenes reference database.

**Figure 2:**
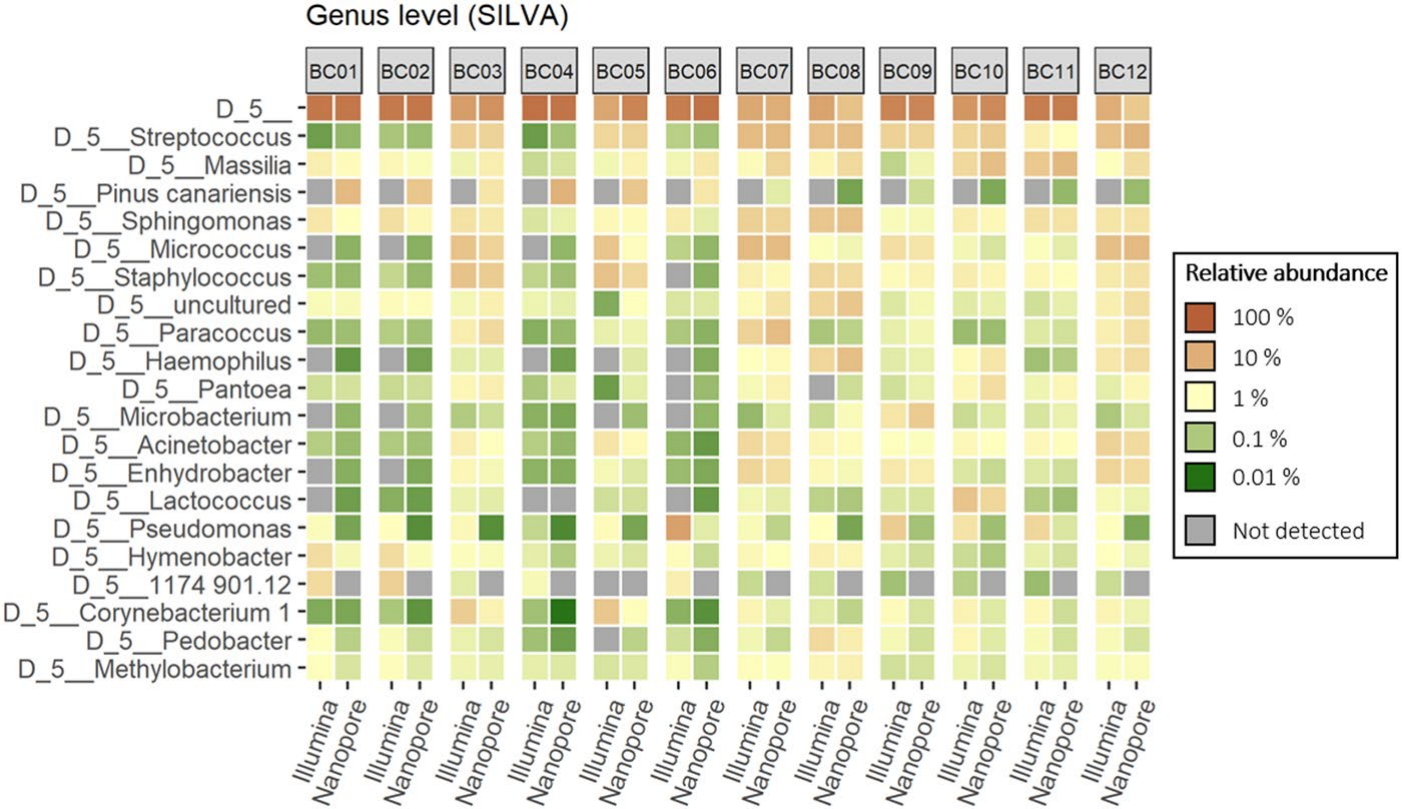
Heatmap of the 15 most abundant genera identified by mapping 16S rRNA gene amplicons sequenced on Illumina MiSeq and Nanopore MinION against the SILVA reference database.

Both short-read amplicons sequenced by Illumina MiSeq and long-read amplicons sequenced by Nanopore MinION were taxonomically assigned against the GG and SILVA databases. The microbial classifications obtained were compared at different taxonomic levels (order, family, genus, and species) for all 12 samples. The relative abundance of the 15 most abundant taxa determined at genus and species level with each platform are shown using heatmaps in Figs. 1, 2. 3 and 4. Heatmaps for order and family level are shown in Supplement 1–4.

**Figure 3:**
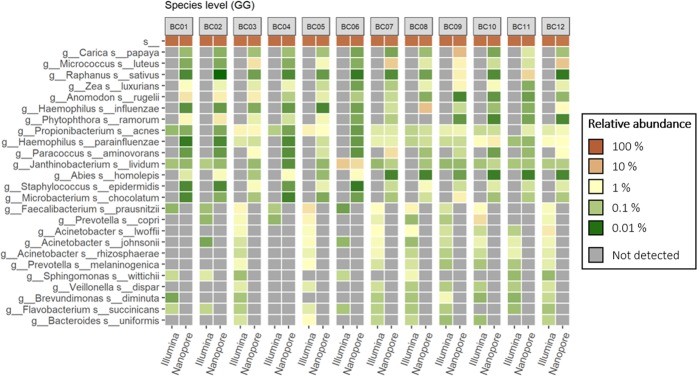
Heatmap of the 15 most abundant species identified by mapping 16S rRNA gene amplicons sequenced on Illumina MiSeq and Nanopore MinION against the Greengenes reference database.

**Figure 4:**
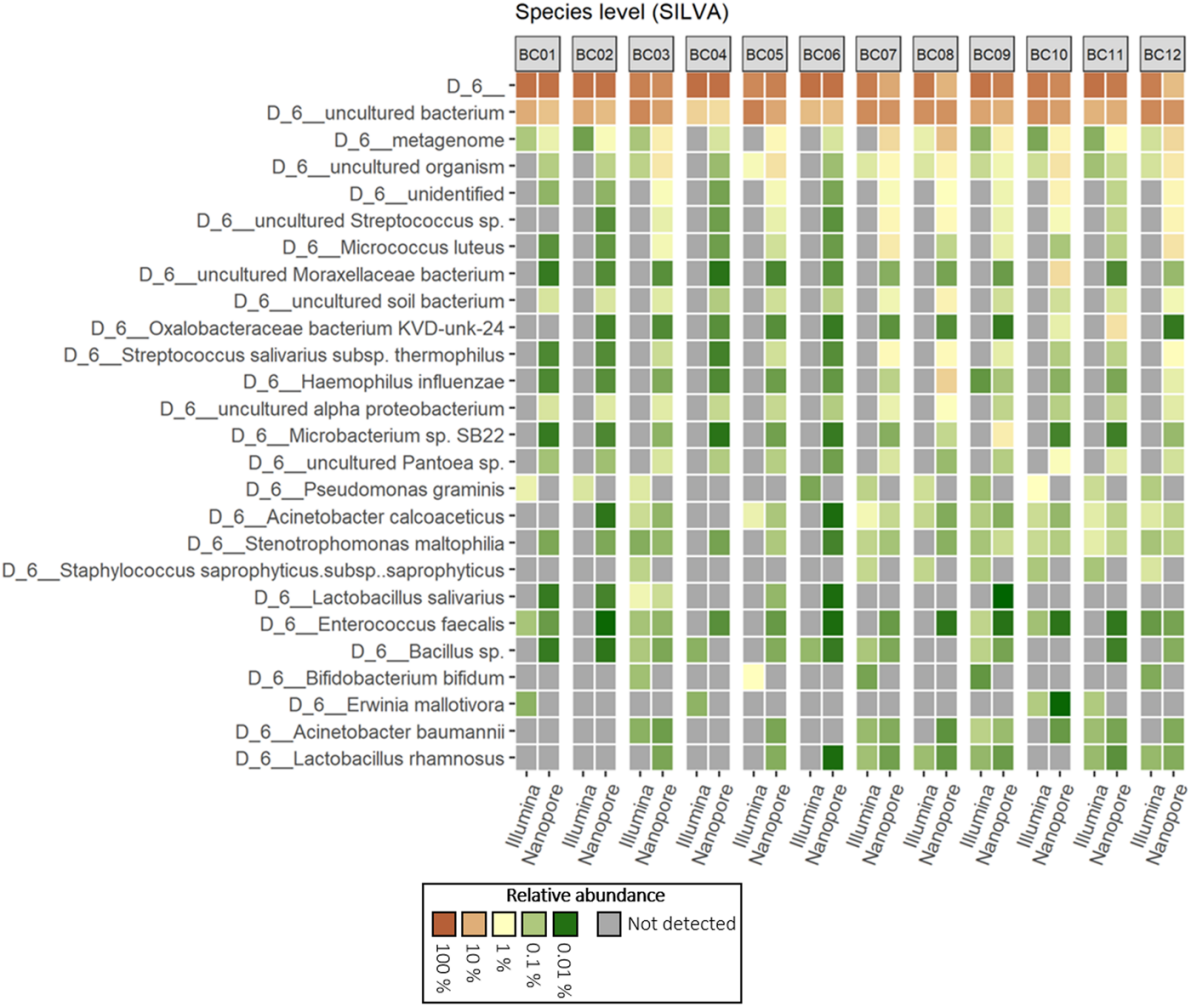
Heatmap of the 15 most abundant species identified by mapping 16S rRNA gene amplicons sequenced on Illumina MiSeq and Nanopore MinION against the SILVA reference database.

At the species level only a few taxa were identified by *both* long-read and short-read sequences (Table 3, Figs. 3 and 4). This is most notable for alignments against the SILVA database, where most of the taxa were identified only by the long-read sequencing platform, e.g. *Micrococcus luteus, Streptococcus salivarius* subsp. *thermophilus* and *Haemophilus influenzae*. The opportunistic pathogen *Stenotrophomonas maltophilia* was identified at low relative abundance across all samples but only when using the SILVA database. Species-level assignments also reveal signature differences between intake and indoor samples. The commensal *M. luteus*, although identified in all samples using both databases, is indicated at consistently higher relative abundances in samples originating in the indoor space, particularly floor dust. A somewhat similar trend, again only revealed by long read sequencing, was found for the nasopharynx commensal *Haemophilus influenza*. In almost every instance, only long read sequences were able to indicate the presence of these species (Figs. 3 and 4).

At the genus level, both GG and SILVA alignments showed that the short-read Illumina amplicons gave higher relative abundancies of *Pseudomonas* in all samples. Samples from outdoor sources (BC01, BC02, BC04, and BC06) showed the largest differences between long and short reads.

As Silva and GG performed somewhat differently in assigning long-read amplicons, the dataset was also analysed using BLAST against the NCBI 16S rDNA database. The curated NCBI 16S database contains approximately 20 000 sequences, compared to the 190 000 and 99 000 sequences in the full SILVA and Greengenes databases, respectively. Table 4 illustrates the most abundant taxa at all sample sites using all three databases, GG, SILVA and NCBI. The results with NCBI were most similar to those obtained with GG. Samples from heating, ventilation and air conditioning (HVAC) exhaust filter dust (BC03, BC05, BC07, and BC12) and floor dust (BC08-BC11) had a higher abundance of genera associated with human activity (e.g. *Streptococcus*, *Micrococcus*, *Staphylococcus*, *Corynebacterium*) (Table 4, Figs. 1 and 2). Conversely, genera commonly found in soil and water (e.g. *Janthinobacterium*, *Hymenobacter*, *Pedobacter*) were generally abundant in samples BC01, BC02, BC04, and BC06, which were intake air dust samples originating from outdoor sources (Table 4, Figs. 1 and 2).

**Table 4:** The most abundant taxa at the genus level identified from the three different sample types using Illumina short-read sequences and Nanopore long-read sequences and three different databases.

Sample type	Illumina (short-read sequencing)				Nanopore (long-read sequencing)					
	GG	%	SILVA	%	GG	%	SILVA	%	NCBI	%
Floor dust	NA^a^	18,45	NA	12,64	NA	17,71	NA	12,83	NA	16,43
	Streptococcus	1,35	Streptococcus	1,35	Streptococcus	1,47	Massilia	1,68	Streptococcus	1,98
	Pseudomonas	0,96	Pseudomonas	0,96	Janthinobacterium	1,15	Streptococcus	1,49	Massilia	1,14
	Sphingomonas	0,87	Sphingomonas	0,91	Sphingomonas	0,85	Sphingomonas	0,92	Sphingomonas	1,07
	Staphylococcus	0,61	Massilia	0,84	Haemophilus	0,82	Haemophilus	0,84	Moraxella	0,82
	Lactococcus	0,56	Staphylococcus	0,64	Carica	0,74	uncultured	0,62	Microbacterium	0,78
	Pedobacter	0,50	Lactococcus	0,56	Staphylococcus	0,54	Staphylococcus	0,58	Staphylococcus	0,72
	Micrococcus	0,43	Pedobacter	0,50	Microbacterium	0,49	Microbacterium	0,55	Haemophilus	0,61
	Chryseobacterium	0,43	Micrococcus	0,44	Raphanus	0,45	Pantoea	0,41	Lactococcus	0,45
	Haemophilus	0,43	Chryseobacterium	0,43	Moraxella	0,38	Eutrema salsugineum	0,40	Acinetobacter	0,44
HVAC exhaust filter	NA	10,79	NA	5,40	NA	14,30	NA	8,01	NA	13,15
	Micrococcus	2,25	Micrococcus	2,36	Streptococcus	2,24	Streptococcus	2,25	Streptococcus	2,75
	Streptococcus	1,93	Streptococcus	1,93	Micrococcus	1,54	Micrococcus	1,80	Micrococcus	1,52
	Staphylococcus	1,32	Staphylococcus	1,39	Paracoccus	1,17	Paracoccus	1,20	Paracoccus	1,28
	Corynebacterium	1,20	Corynebacterium	1,10	Staphylococcus	0,97	Staphylococcus	1,00	Staphylococcus	1,21
	Acinetobacter	0,93	Acinetobacter	0,93	Sphingomonas	0,73	Massilia	0,82	Sphingomonas	0,83
	Prevotella	0,85	Sphingomonas	0,85	Acinetobacter	0,63	Sphingomonas	0,78	Moraxella	0,72
	Bacteroides	0,85	Bacteroides	0,85	Enhydrobacter	0,60	Pinus canariensis	0,69	Acinetobacter	0,60
	Enhydrobacter	0,80	Enhydrobacter	0,80	Exiguobacterium	0,45	uncultured	0,64	Massilia	0,57
	Sphingomonas	0,78	Lactobacillus	0,76	Haemophilus	0,36	Acinetobacter	0,64	Exiguobacterium	0,45
HVAC intake filter	NA	27,79	NA	23,94	NA	29,79	NA	25,55	NA	28,43
	Pseudomonas	1,78	Pseudomonas	1,78	Janthinobacterium	0,54	Pinus canariensis	2,32	Janthinobacterium	0,58
	Hymenobacter	0,61	1174 901.12	0,81	Anomodon	0,35	Picea glauca (white spruce)	0,44	Sphingomonas	0,53
	Sphingomonas	0,55	Hymenobacter	0,61	Phytophthora	0,31	Janthinobacterium	0,39	Methylobacterium	0,28
	Janthinobacterium	0,47	Sphingomonas	0,58	Abies	0,27	1174–901–12	0,39	Massilia	0,25
	Methylobacterium	0,22	Janthinobacterium	0,45	Sphingomonas	0,26	Massilia	0,36	Cylindrospermum	0,20
	Pedobacter	0,17	Massilia	0,32	Zea	0,17	Sphingomonas	0,27	Stanieria	0,15
	Erwinia	0,13	Acidiphilium	0,30	Hymenobacter	0,16	uncultured	0,23	Oscillatoria	0,14
	Buchnera	0,12	Methylobacterium	0,28	Buchnera	0,09	Dicranaceae sp. Goffinet 11067	0,22	Gluconacetobacter	0,11
	Flavobacterium	0,12	uncultured	0,24	Methylobacterium	0,09	Hymenobacter	0,16	Granulicella	0,10

^a^Not assigned taxonomies.

### Long-read and short-read sequencing correlation

**Figure 5:**
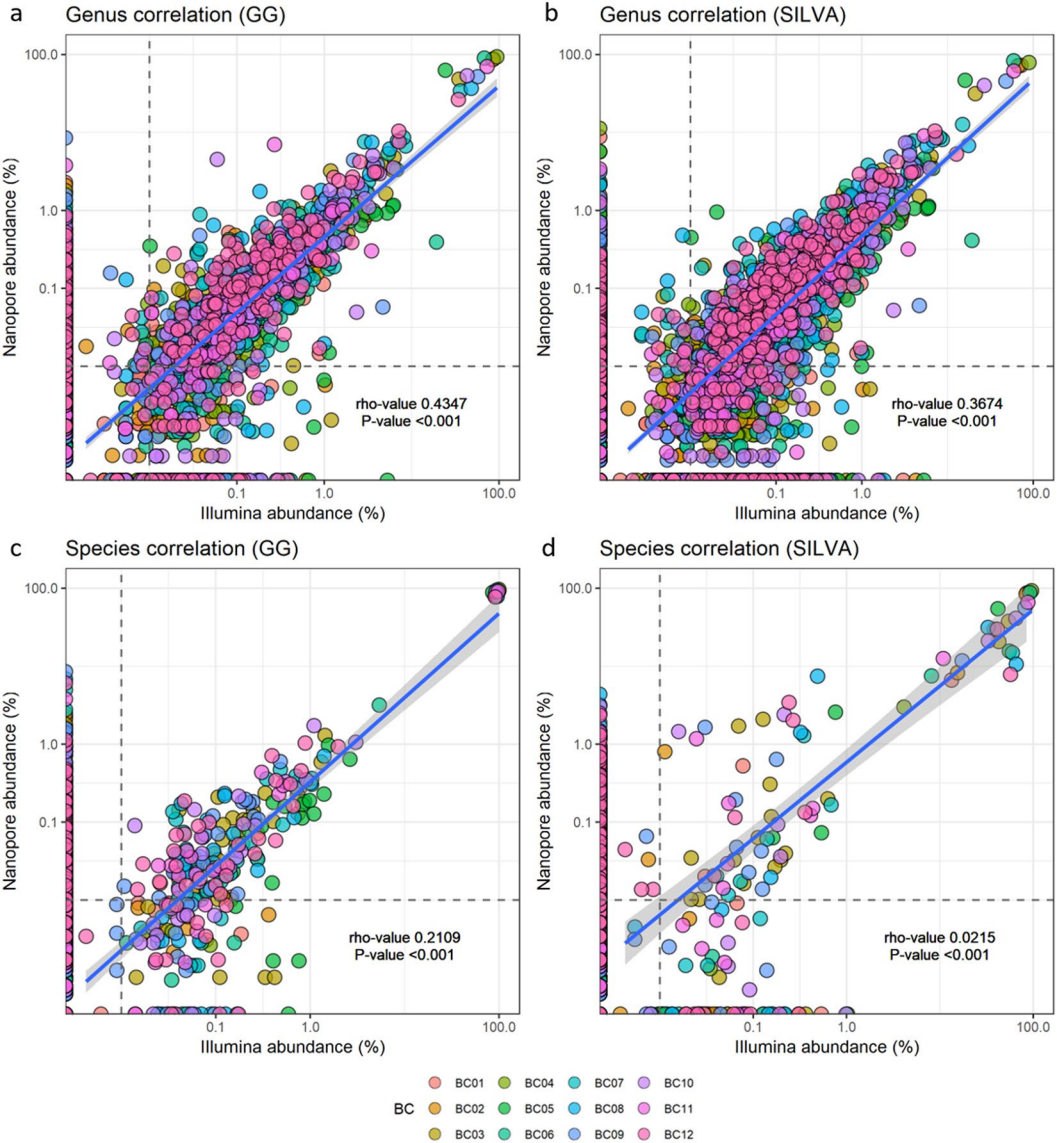
Correlation of identified taxa at (**a**) the genus level against GG, (**b**) genus level against SILVA, (**c**) species level against GG, and (**d**) species level against SILVA between sequencing platforms for all 12 samples. The dashed lines mark a 0.01% relative abundance threshold for each taxa for Nanopore and Illumina sequence data.

Spearman’s rank correlation illustrated that the sequencing platforms revealed similar bacterial composition at the level of order and family, while the results at the genus and species levels differed to a higher degree for some samples (Fig. 5, Supplement 5–12).

Analysis of individual samples showed a strong or moderate positive correlation between the sequencing platforms at the order level for all samples (Supplement 5 and 9). At the family level, eight samples had a strong positive correlation between the sequencing platforms when aligned against GG, whereas eight samples had a moderate positive correlation (Supplement 6). When aligned against SILVA, two samples had a moderate positive correlation (BC08 and BC12), and six samples had a weak positive correlation. The remaining samples had either a negligible or non-significant correlation (Supplement 10). At the genus level, the results obtained with long and short-reads against GG showed a moderate positive correlation for samples BC03 and BC07. For the remaining samples, the correlations were either a negligible or non-significant (Supplement 6). All samples had either a neglible or non-significant correlation at the genus level when aligned against SILVA (Supplement 11). At the species level, all samples had either a negligible or non-significant correlation between the sequencing platforms, when aligned against both GG and SILVA. (Supplement 8 and 11)

In the correlation plot of the identified taxa (Fig. 5) it can be seen that a larger proportion of the Nanopore sequences fall below 0.01% abundance compared to Illumina sequences. This is seen at both the genus and species level for identifications against both GG and SILVA.

## Discussion

We analyzed 16S rRNA gene amplicons generated from 12 dust samples collected from kindergartens and nursing homes in Norway. Two types of sequencing libraries were prepared: Short-read amplicons for sequencing on Illumina MiSeq were prepared by amplifying the V3-V4 hypervariable regions (approximately 464 bp) of the 16S rRNA gene. Long-read amplicons for sequencing on Nanopore MinION covered the V1-V9 hypervariable regions (approximately 1465 bp), making up nearly the full length of the 16S rRNA gene.

Because of the different read length capabilities of the two sequencing platforms, different regions and different primer pairs were used for Nanopore MinION and Illumina MiSeq sequencing. The 16S rRNA regions are variably informative, and the region analyzed is, therefore, likely to affect the taxonomic outcome. Soergel *et al*.^13^ computed the classification rate for 374 pairings of 22 forward primers and 22 reverse primers for 16S rRNA and read lengths across different environments. They found that primer choices greatly affect taxonomic informativeness and that the most informative primers differed with respect to the material under investigation. For dust and skin samples, primer 1492R combined with 341F, was shown to produce robust predictions at the genus level^13^. In the present study, the primer pair 1492R/27F was used for the MinION procedure. The Illumina analyses were performed by a commercial laboratory which routinely uses the primer pair 341F/805R.

An additional factor long known to affect taxonomic classifications is the choice of reference databases, as the number and origins of reference sequences included in different databases varies greatly^14^.

Since few microbiome-studies exist with full-length 16S rRNA sequences, the genus level is commonly used for comparison of samples or environments. The major genera identified in the present study are in general agreement with previously published works on indoor dust microbiomes^15–17^. Both long and short read sequences when accessed against the databases used in this study revealed the same signature differences between the bacterial content of outdoor and indoor samples – i.e., a relative preponderance of taxa associated with human activity in the latter. Furthermore, both sequencing platforms (including here primer choice) resulted in similar taxonomic classifications for all samples at the order and family level. Both platforms performed similarly for samples originating from the indoor environment (i.e. HVAC exhaust and floor dust samples) whereas samples of outdoor origin (i.e. HVAC intake samples) manifest greater differences between the sequencing platforms. Thus, either approach could be used where the aim is to reveal the major structural differences in bacterial content of the indoor and outdoor spaces.

However, at the genus and particularly species levels, some key differences emerge in the datasets with respect to the sequencing technologies used and the databases accessed. The MinION platform, which provided nearly full-length 16S rRNA gene sequences, gave a significantly higher resolution at the species level (Table 3). A number of species were identified only with long-read sequences (Figs. 3 and 4), suggesting that a partial sequence region of the 16S rRNA gene cannot provide the same taxonomic resolution as full-length sequences^18^. This is in line with Shin *et al*. who compared the mouse microbiome as revealed by the same two sequencing platforms^19^. Taken together, these two studies suggest that MinION may be able to provide high taxonomic resolution of fundamentally different microbiomes. However, some studies show that analysis of the whole *rrn* operon (16S rRNA–ITS–23S rRNA) represents a more powerful tool than analysis of merely the 16S rDNA gene for resolution of taxa at the species level^20^. Basing their analyses on the *rrn* operon, Cusco *et al*.^16^ were able to delineate a greater number of species in the sequence data, further illustrating the limitations of the 16S rDNA alone in species allocation^20,21^. Identification to the species level is important not only because it provides a more detailed description of the microbial communities of interest, but also because pathogenicity is usually a species or strain level phenomenon^22^. For example, some species of potentially medical importance were only identified using long read sequences and only with one or another database. *S. maltophilia* was only detected when matching long, and for some samples short sequences, against the SILVA database (Fig. 4). *S. maltophilia* is an environmental opportunistic pathogen. The incidence of nosocomial and community-acquired infections (particularly respiratory) of immunocompromised individuals caused by this species, is an increasing concern^23^. Furthermore, only short-read Illumina sequences when accessed against the SILVA database produced a species-level identification for a member of the genus *Pseudomonas*. The genus *Pseudomonas* houses some opportunistic human-pathogenic-species, most especially *P. aeruginosa*. However, particularly when drawing conclusions concerning genus and species level identification using sequencing, one has to consider the risk of wrongly assigned taxonomies. The use of reference databases that contain larger numbers of sequences could increase the risk of false positive identifications. The most widely used databases in similar studies are Greengenes and SILVA, as these are included in many of the commonly used piplines for analysis of 16S rRNA sequencing data. Therefore, although more limited in terms of the number of sequences, the highly curated NCBI 16S rRNA database was also included to assigned taxonomies at the genus level (Table 4). The results with NCBI are most similar to those obtained with Greengenes, providing support for the continued use of the latter.

## Conclusion

Results for 16S rRNA amplicon analysis obtained with MinION are promising. Oxford Nanopore’s long-read chemistry could make species level identification of the bacteria comprising building-dust microbiomes more accessible, thus improving classifications of these bacterial communities. The present study is to our knowledge the first attempt to investigate the indoor microbiome using the Nanopore MinION sequencing technology. We demonstrate that species level identification may be possible, which could be useful when studying potential routes of disease transmission in the indoor space. However, more comprehensive analyses using a larger number of replicates are required to confirm the suggestions put forth in this paper. The low sampling volume provides an insufficient number of biological replicates to make accurate profiles of the dust microbiomes. Following on, it would also be useful to analyze larger data sets with additional, curated rRNA genes databases to see if these reveal similar structures to those presented here, or if new details emerge.

## Methods

### Samples

Building dust samples were collected from kindergartens and nursing homes in Norway. Samples BC01-BC05 (Table 5) are dust samples collected from HVAC filters from HVAC units located in nursing homes. Samples BC06, BC07, and BC12 are collected from HVAC filters in kindergartens. Samples BC08-BC11 are floor dust samples collected from a kindergarten. HVAC filter dust samples were collected as described in Nygaard and Charnock^15^. Procedures for sampling of floor dust samples were as given in Nygaard and Charnock^24^.

**Table 5:** Sample identification, description and origin.

Sample ID	Building type	Dust sample type
BC01	Nursing home	HVAC intake filter
BC02	Nursing home	HVAC intake filter
BC03	Nursing home	HVAC exhaust filter
BC04	Nursing home	HVAC intake filter
BC05	Nursing home	HVAC exhaust filter
BC06	Kindergarten	HVAC intake filter
BC07	Kindergarten	HVAC exhaust filter
BC08	Kindergarten	Floor dust
BC09	Kindergarten	Floor dust
BC10	Kindergarten	Floor dust
BC11	Kindergarten	Floor dust
BC12	Kindergarten	HVAC exhaust filter

### DNA extraction

DNA was extracted from approximately 100 mg dust from each sample using the PowerWater DNA isolation kit (MO BIO, CA, USA) as previously described by Nygaard *et al*.^15^. DNA concentrations were measured using Qubit 3.0. fluorometer and Qubit dsDNA HS Assay kit (Thermo Fisher Scientific, Waltham, MA, USA).

### Sequencing

#### Long-read 16S Nanopore sequencing

Five ng DNA from each sample were used in PCR reactions with 16S primers 27 F and 1492 R (MWG Eurofins, GmBh) for amplification of the near full-length bacterial 16S rRNA gene (Table 6). Amplicons (800 ng) from each sample were end repaired and dA-tailed using NEBNext End-Repair and NEBNext dA-Tailing modules (New England Biolabs) according to the manufacturer’s instructions. Using the 1D Native barcoding genomic DNA kit EXP-NBD103, R9 version (Oxford Nanopore Technologies, Oxford, UK) barcodes were ligated to the dA-tailed DNA using Blunt/TA Ligase Master Mix (New England Biolabs). Then sequencing adapters were ligated to the pooled barcoded reads according to the manufacturer’s instructions using sequencing kit 1D SQK-LSK108, R9 version (Oxford Nanopore Technologies) to complete the library building. Sequencing was performed using a FLO-MAP R7.3 flowcell for 48 hours on the MinION portable sequencer (Oxford Nanopore Technologies). Nanopore sequence data are deposited in the European Nucleotide Archives (ENA) and is available through accession numbers ERS2702700-ERS2702711.

#### Short-read 16S Illumina Miseq sequencing

DNA from the same samples was sent to a commercial laboratory, Omega Bioservices (Atlanta, Georgia, USA), for 2 × 300 bp paired-end sequencing. The libraries were prepared using Illumina 16S Metagenomic Sequencing kit (Illumina, Inc., San Diego, CA, USA) according to the manufacturer’s protocol. The V3-V4 region of the bacterial 16S rRNA gene sequences was amplified using the primer pair 341F-805R, containing the gene‐specific sequences and Illumina adapter overhang nucleotide sequences. Primer sequences are shown in Table 6. Illumina sequence data has been deposited in the ENA and is available through accession numbers ERS2702688-ERS2702699.

**Table 6:** Primers used for generating short-read and long-read amplicons.

Primer set	Primer name	16S Region	Sequence	Reference
Long-read amplicons (MinION sequencing)	27F 1492R	V1-V9	5′ AGAGTTTGATCMTGGCTCAG 3′ 5′ TACGGYTACCTTGTTACGACTT 3′	Weisburg, *et al*.^30^
Short-read amplicons (Illumina sequencing)	341F 805R	V3-V4	5′ CCTACGGGNGGCWGCAG 3′ 5′ GACTACHVGGGTATCTAATCC 3′	Herlemann, *et al*.^31^

### Sequence analysis

#### Taxonomic reference databases

After sequence data processing (described below) both long- and short-read amplicons were taxonomically assigned using the GG 13_8 97% reference sequences^25^ and the SILVA 132 99% reference sequences. In addition, long-read amplicons were taxonomically assigned using the NCBI 16S rDNA database.

#### Long-read 16S sequencing data processing, taxonomic assignment and analysis

Raw fast5 reads were basecalled, sorted by their respective barcodes and converted to fastq files using Albacore (version 2.1.10). Sequencing adapters were removed using Porechop (version 0.2.3) (https://github.com/rrwick/Porechop) and the trimmed sequences quality filtered using NanoFilt (version 1.8.0) (https://github.com/wdecoster/nanofilt). Sequences were filtered on a minimum average read quality score, and only sequences with an average quality score of 9 or above were retained. Resulting fastq files were converted to fasta using Fastx-Toolkit. The trimmed and quality filtered reads were then aligned against the GG 13_8 97% reference sequences^25^ and the SILVA 132 99% reference sequences using the LAST aligner (v.921) (http://last.cbrc.jp/) with the following parameters: -r 1 -q 1 -a 1 -b 1 (match score of 1, mismatch cost of 1, gap opening cost of 1, and gap extension cost of 1). For each read, the highest scoring alignment was retained and assigned with the taxonomic id of the corresponding GG reference sequence. Taxonomic IDs with only one aligned sequence read were discarded from the sample.

The basecalled long-read 16S-sequences were also taxonomically assigned using the cloud-based EPI2ME Fastq 16S workflow provided by Nanopore. Here, basecalled sequences are mapped against the NCBI 16S bacterial database using BLAST. After that, each read is classified based on % coverage and identity.

#### Short-read 16S sequencing data processing, taxonomic assignment and analysis

Demultiplexed paired-end fastq files and a mapping file were used as input files. Sequences were pre-processed, quality filtered and analyzed using QIIME2 (2018.2 release) (https://qiime2.org/). DADA2^26^ in QIIME2 was used for sequence correction and removal of chimeras. Paired sequence reads were joined and quality-filtered using the paired-end DADA2 pipeline, using default settings. Primers were trimmed using the –p-trim-left function. The forward reads were truncated to 290 bases and the reverse reads to 200 bases, allowing for an overlap of 25 bases in merged sequences. To generate taxonomy tables, sequences were assigned taxonomies using vsearch^27^ on the GG 13_8 97% reference database^25^ and the SILVA 132 99% reference database. The QIIME2 taxa barplot command was used for viewing the taxonomic composition of the samples and generating abundance data.

#### Statistical analysis

Spearman rank correlation was used to compare the samples microbial community compositions as revealed by the sequencing platforms. Correlations between sequencing platforms were considered to be very strong if Spearmans rho (r_s_) was +/−0.9 to 1, strong if r_s_ was +/−0.7 to 0.9, moderate if r_s_ was +/−0.5 to 0.7, weak if r_s_ was +/−0.3 to 0.5, or neglible if r_s_ was +/−0.0 to 0.3, and if *p* < 0.05^28,29^.

## Supplementary information

Supplementaryinformation

## Data Availability

The raw sequences generated in this project were deposited in the European Nucleotide Archives (ENA) and samples are available through accession numbers ERS2702688-ERS2702711. The Greengenes taxonomy reference sequences analyzed during the current study is available on the Greengenes webpage: ftp://greengenes.microbio.me/greengenes_release/gg_13_5/gg_13_8_otus.tar.gz The SILVA taxonomy reference sequences anayzed during the current study is available in the SILVA file repository: https://www.arb-silva.de/fileadmin/arb_web_db/release_132/ARB_files/SILVA_132_SSURef_NR99_13_12_17_opt.arb.gz The NCBI taxonomy reference sequences analyzed during the current study are deposited in the NCBI database and are available through accession number PRJNA33175.
