# Supplementary material for: A preliminary study on the potential of Nanopore MinION and Illumina MiSeq 16S rRNA gene sequencing to characterize building-dust microbiomes
Source: Sci Rep. 2020 Feb 21;10:3209. doi: 10.1038/s41598-020-59771-0 (PMC7035348; doi:10.1038/s41598-020-59771-0)
Supplement: Supplementary file 1 — Supplementaryinformation [file 41598_2020_59771_MOESM1_ESM.docx]

Supplementary material

A preliminary study on the potential of Nanopore MinION and Illumina MiSeq 16S rRNA gene sequencing to characterize building-dust microbiomes

Anders B. Nygaard*^1,2^, Hege S. Tunsjø^2^, Roger Meisal^3^, Colin Charnock^2^

^1^ Faculty of Technology, Art and Design, Department of Civil Engineering and Energy Technology, Oslo Metropolitan University (OsloMet), Oslo, Norway
^2^ Faculty of Health Sciences, Department of Life Sciences and Health, OsloMet, Oslo, Norway
^3^ Møreforsking Ålesund AS, Ålesund, Norway

*** Corresponding author:**Anders B. Nygaard
Email: anders.b.nygaard@gmail.com

# Supplementary figures


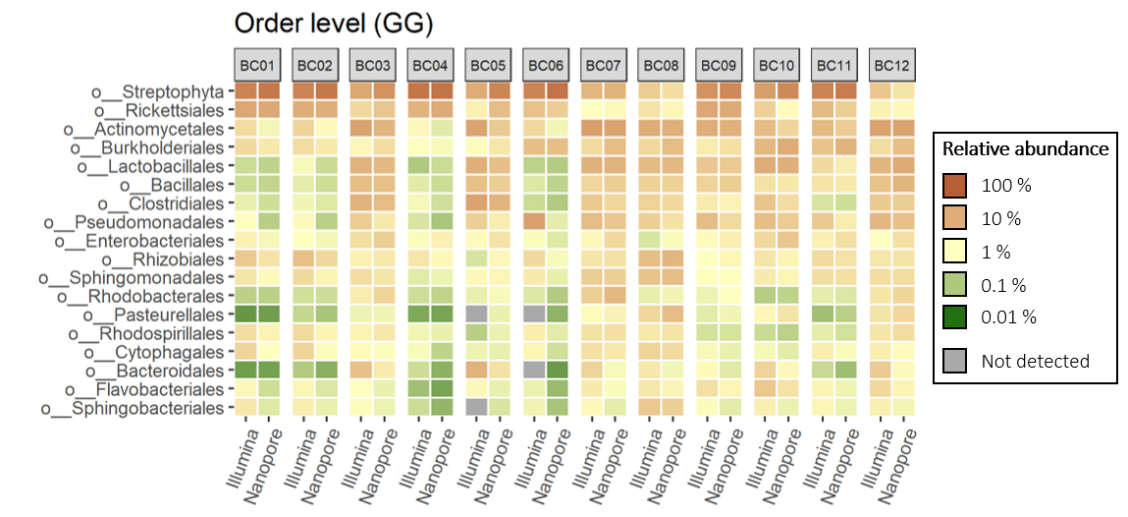


Supplement 1. Heatmap of the 15 most abundant taxa at order level identified by mapping 16S rRNA gene amplicons sequenced on Illumina MiSeq and Nanopore MinION against the GG reference database.


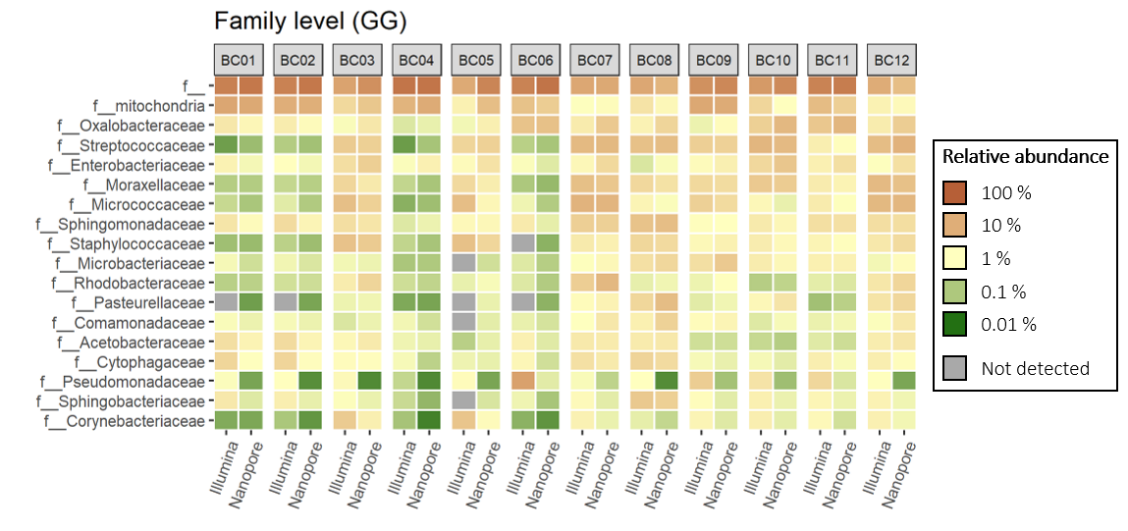


Supplement 2. Heatmap of the 15 most abundant taxa at family level identified by mapping 16S rRNA gene amplicons sequenced on Illumina MiSeq and Nanopore MinION against the GG reference database.


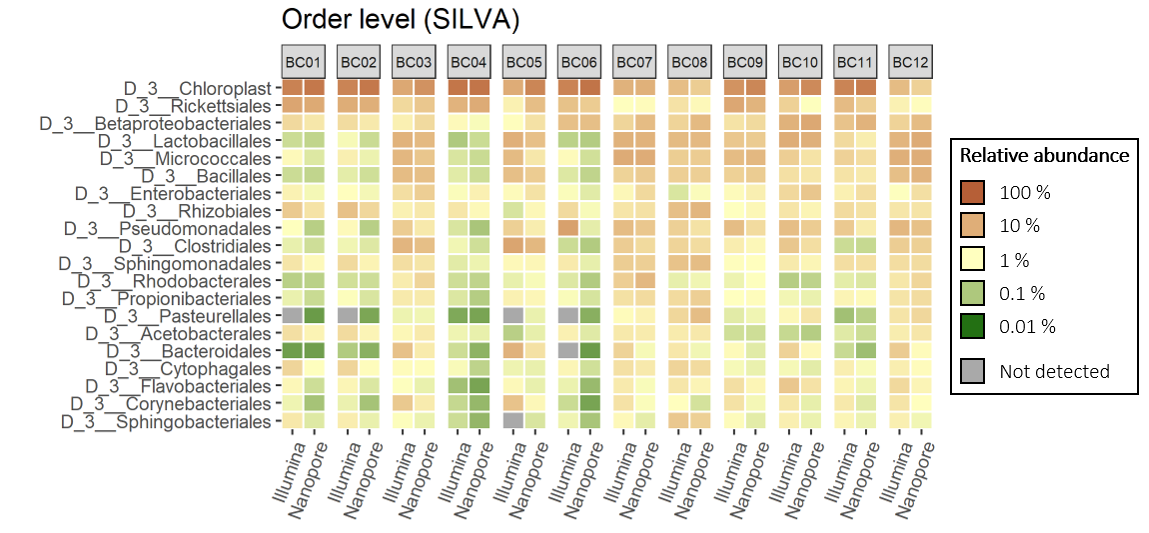


Supplement 3. Heatmap of the 15 most abundant taxa at order level identified by mapping 16S rRNA gene amplicons sequenced on Illumina MiSeq and Nanopore MinION against the SILVA reference database.


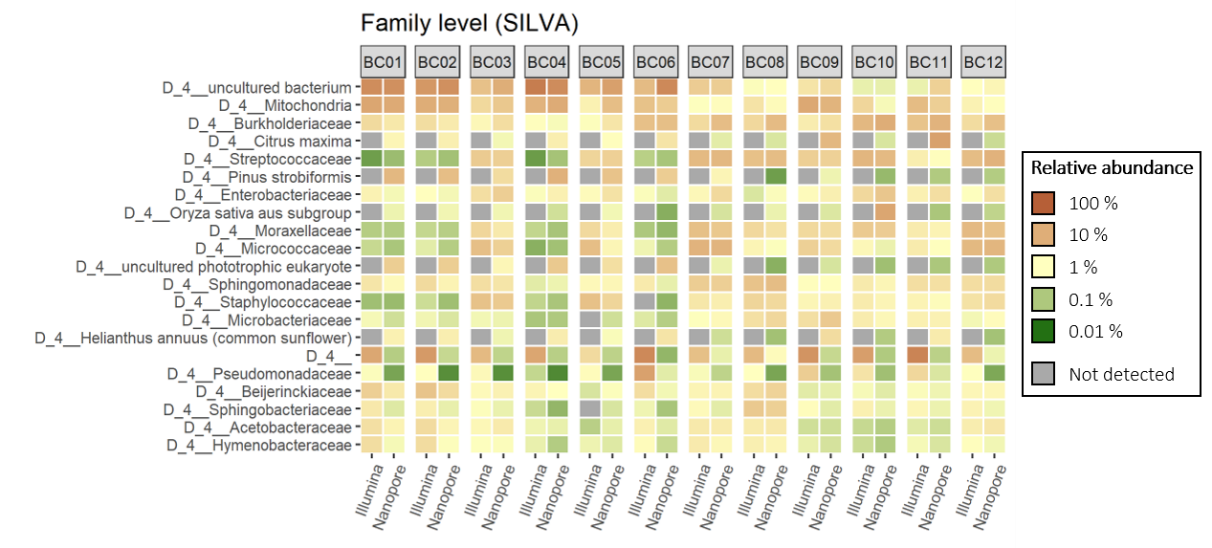


Supplement 4. Heatmap of the 15 most abundant taxa at family level identified by mapping 16S rRNA gene amplicons sequenced on Illumina MiSeq and Nanopore MinION against the SILVA reference database.


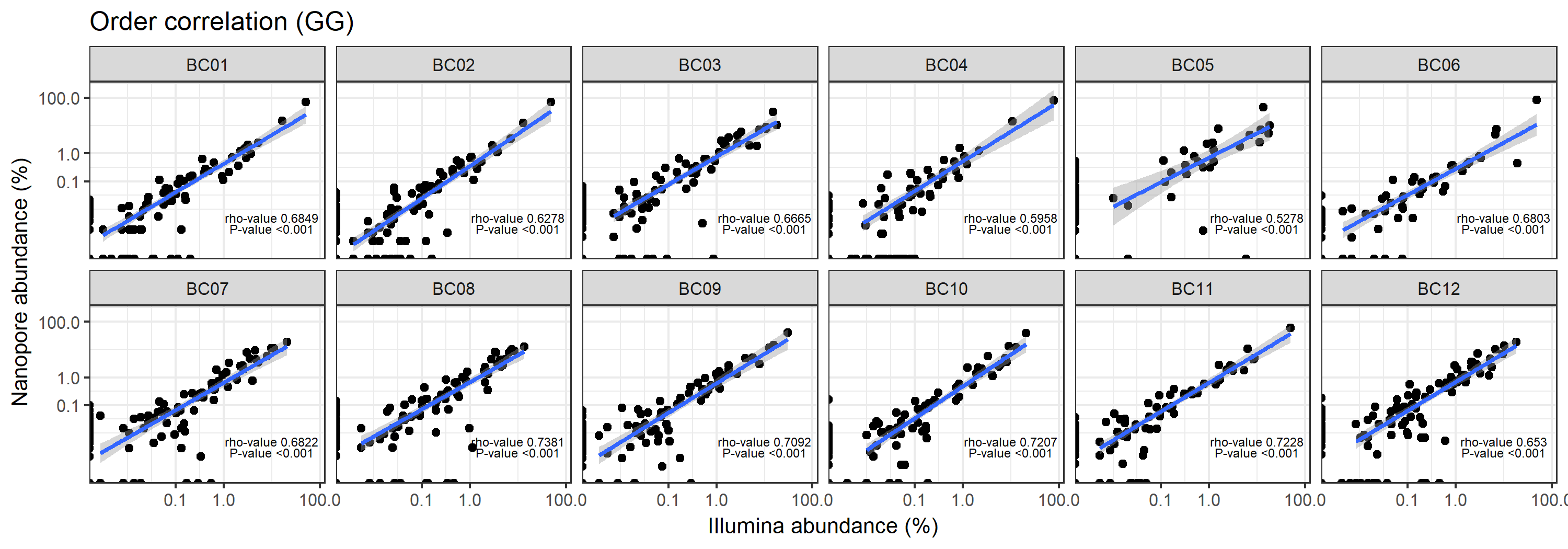


Supplement 5. Order level correlation between Nanopore MinION and Illumina MiSeq sequencing data against Greengenes.


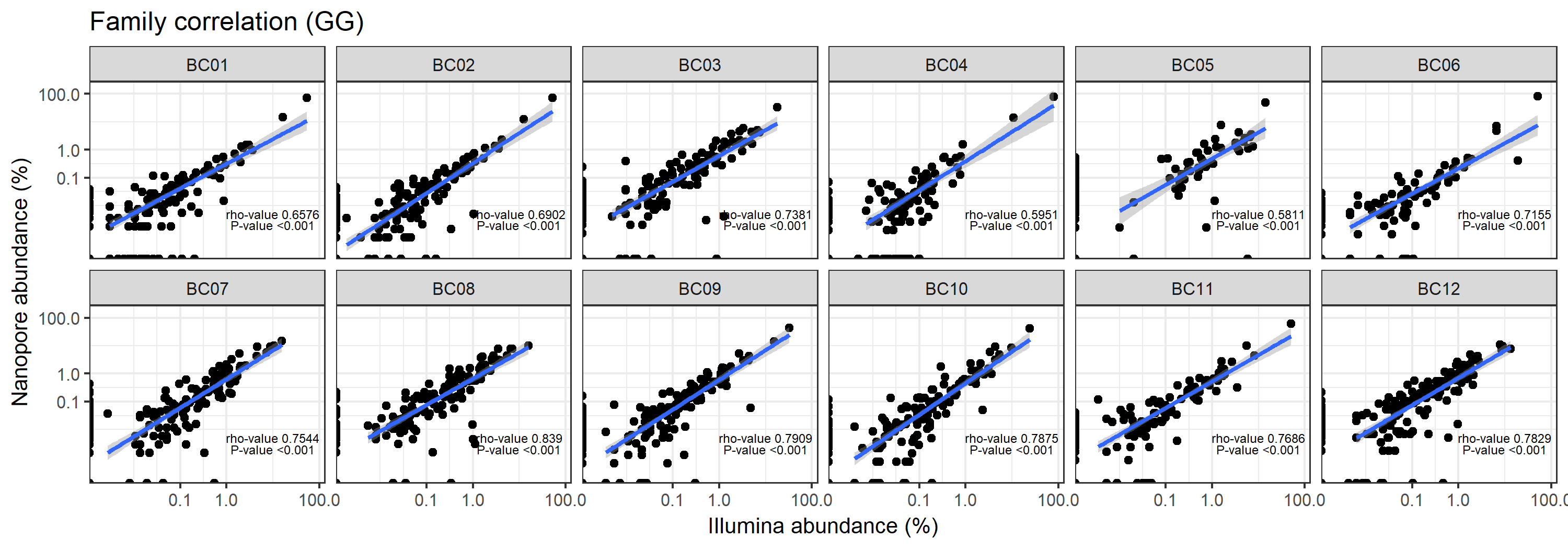


Supplement 6. Family level correlation between Nanopore MinION and Illumina MiSeq sequencing data against Greengenes.


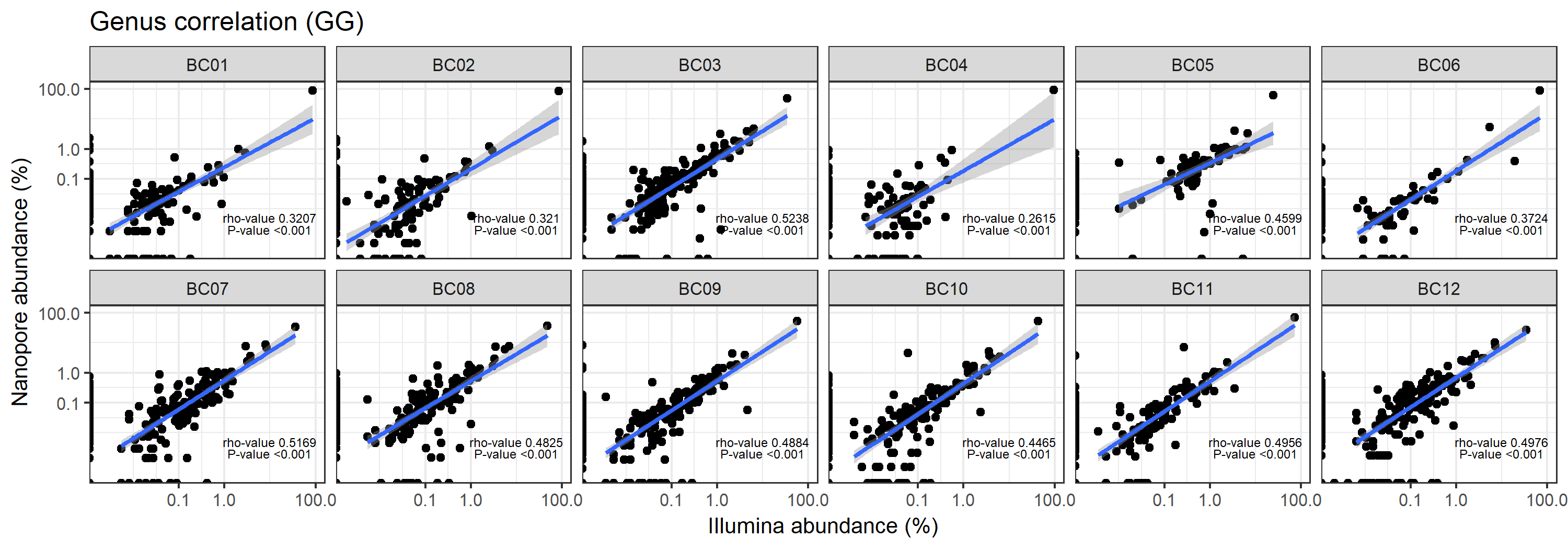


Supplement 7. Genus level correlation between Nanopore MinION and Illumina MiSeq sequencing data against Greengenes.


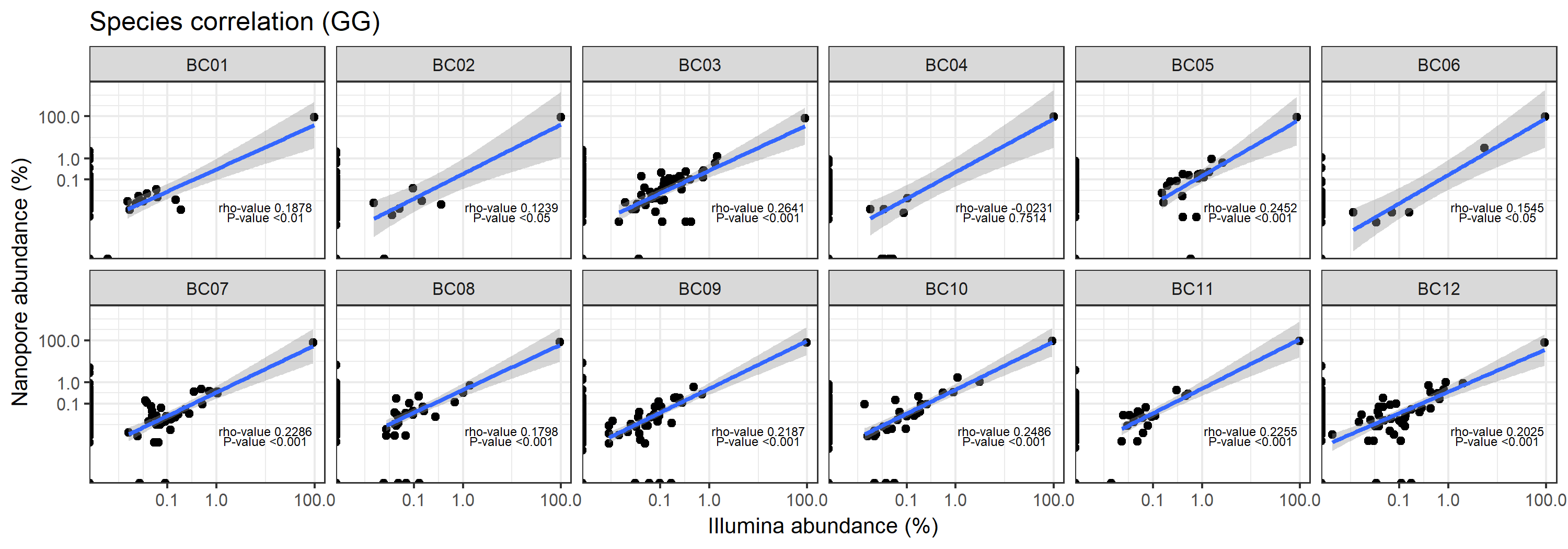


Supplement 8. Species level correlation between Nanopore MinION and Illumina MiSeq sequencing data against Greengenes.


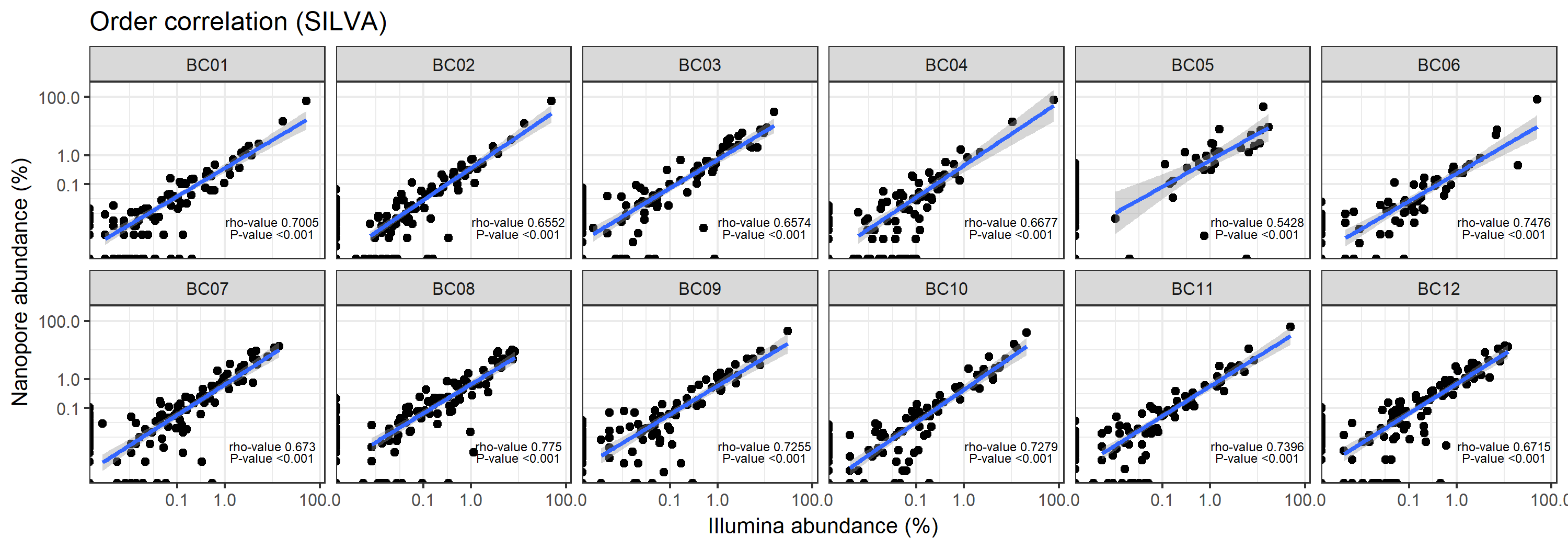


Supplement 9. Order level correlation between Nanopore MinION and Illumina MiSeq sequencing data against SILVA.


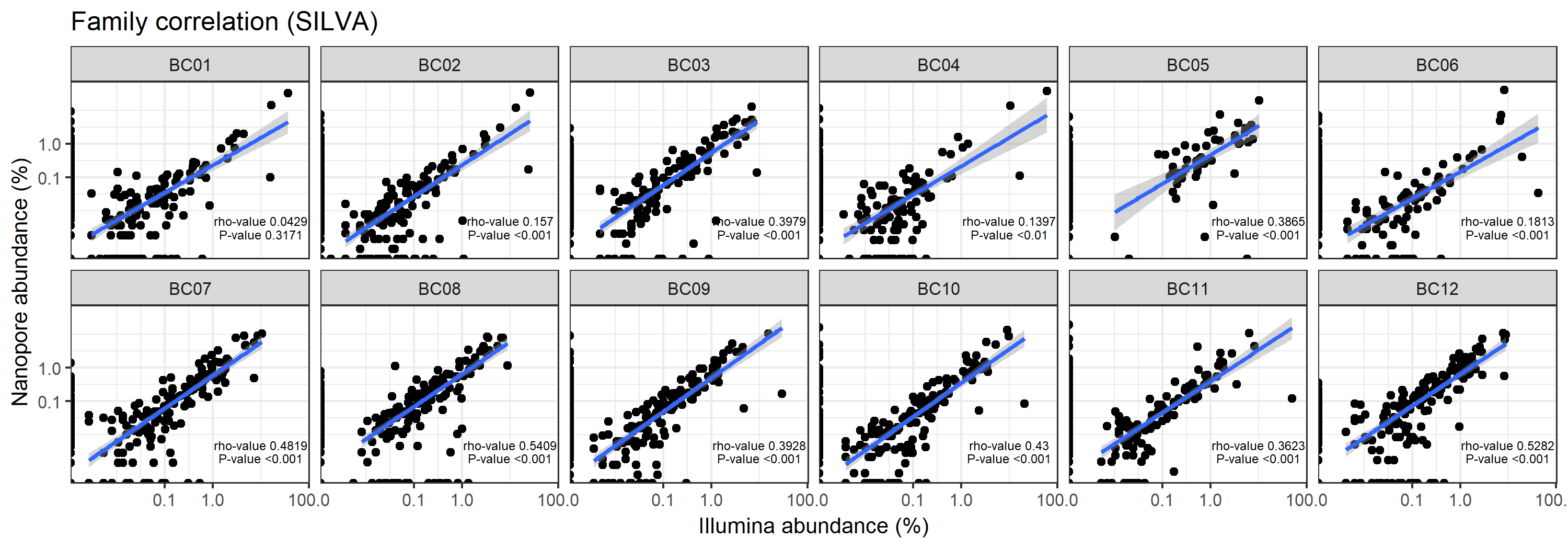


Supplement 10. Family level correlation between Nanopore MinION and Illumina MiSeq sequencing data against SILVA.


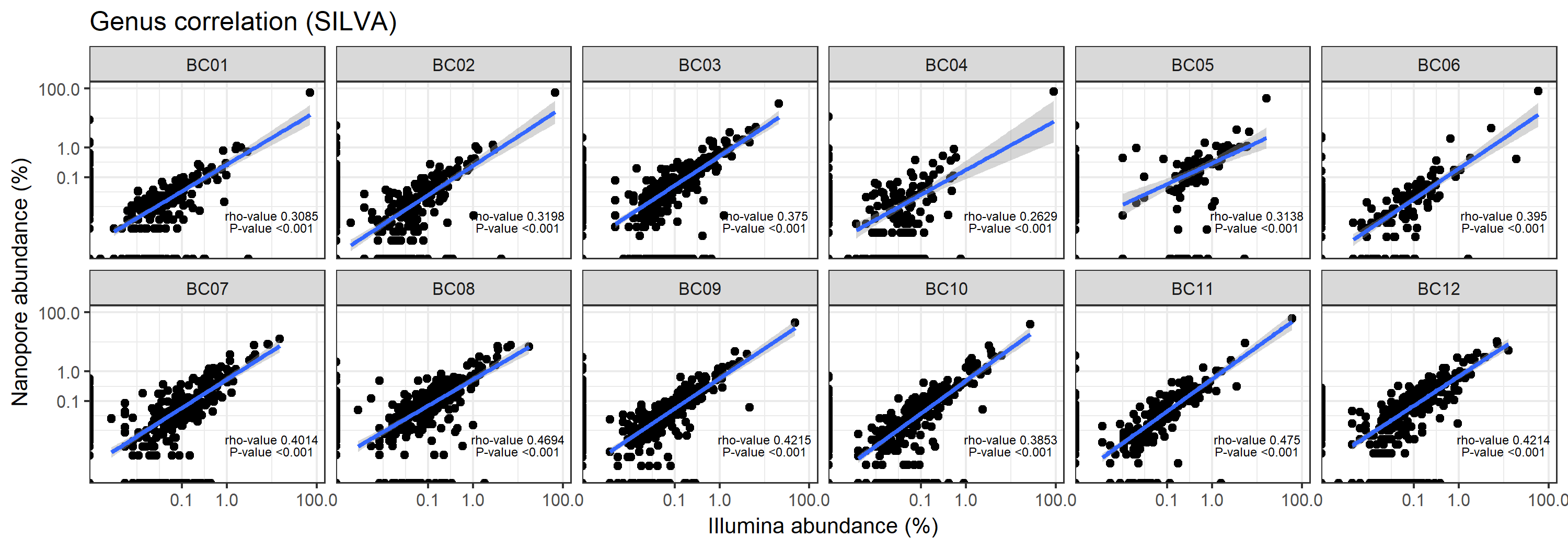


Supplement 11. Genus level correlation between Nanopore MinION and Illumina MiSeq sequencing data against SILVA.


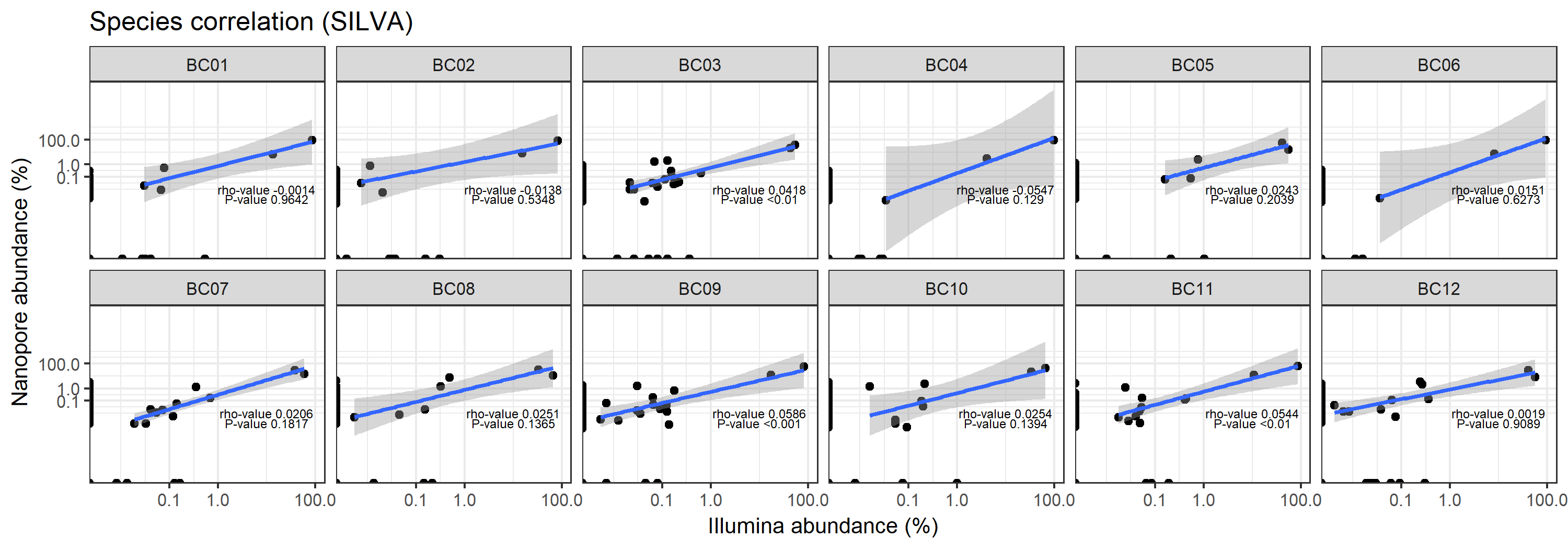


Supplement 12. Species level correlation between Nanopore MinION and Illumina MiSeq sequencing data against SILVA.
